# How can multiple benefit drivers improve corporate ESG performance?—The effects of green technology innovation and peer pressure

**DOI:** 10.1371/journal.pone.0321525

**Published:** 2025-05-19

**Authors:** Bing Ma, Xiaoqi Dong, Peng Shao, Zhen Chen

**Affiliations:** School of Management, Xi’an Polytechnic University, Xi’an, Shaanxi, China; University of Cagliari: Universita degli Studi Di Cagliari, ITALY

## Abstract

To achieve the ‘carbon peaking and carbon neutrality’ objectives, there has been a growing interest in corporate environmental, social, and governance (ESG) performance from governmental and supervisory bodies. This heightened focus has posed critical theoretical and practical questions on how firms can enhance their ESG performance to facilitate their transition to a green economy. From a stakeholder perspective, this study examines data from 724 listed Chinese companies spanning 2015–2021. It aims to elucidate the relationship between benefit drivers (such as media attention, financing constraints, and government subsidies) and corporate ESG performance, along with investigating the mediating role of green technology innovation and the moderating influence of peer pressure in this context. The findings suggest that both media attention and government subsidies positively correlate with ESG performance, while financing constraints exhibit a negative correlation. Moreover, green technology innovation serves as a mediator between benefit drivers and corporate ESG performance. Furthermore, peer pressure can serve as a positive moderator in the relationship between media attention and green technology innovation, as well as in the negative relationship between financing constraints and green technology innovation. Conversely, it can negatively moderate the positive relationship between government subsidies and green technology innovation. Heterogeneity analysis reveals that in enterprises with superior resource acquisition capabilities, benefit drivers exert a more pronounced influence on corporate ESG performance. Similarly, in state-owned enterprises, benefit drivers also have a heightened impact on corporate ESG performance.

## 1. Introduction

Corporate Environmental, Social, and Governance (ESG) performance has increasingly become a significant investment concept and corporate action guide in the financial market. This is because it provides an accurate measure of a company’s sustainable development capacity, facilitating the coordinated progression of the economy, society, and environment. As environmental issues such as air pollution and climate change intensify, many countries have recognized the necessity of achieving green development and ensuring a harmonious relationship between humankind and nature. In this context, corporate ESG performance offers an effective pathway toward the advancement of a green economy. However, there are still no established standards for enhancing corporate ESG performance. Consequently, identifying ways to effectively improve corporate ESG performance is a critical issue that requires immediate attention.

Prior research has delved into the antecedent variables of corporate ESG performance, focusing on internal governance structures and external environmental factors such as directors’ compensation [1] and industry sensitivity [2]. However, these studies have overlooked the motivational drivers from stakeholder perspectives. While a few studies have indicated that government financial subsidies can stimulate corporate enthusiasm and prompt fulfillment of social and other ESG responsibilities [3], a systematic exploration of the impact mechanisms and improvement strategies related to interest-driven factors like media attention, financing constraints, and government subsidies from a stakeholder perspective remains notably limited.

Stakeholder theory suggests that the engagement and contributions of key stakeholders, such as media, investment institutions, and governments, significantly impact corporate governance and operational activities. This engagement subsequently drives companies to enhance their ESG performance and sustainable development capabilities. On one hand, these stakeholders contribute to a company’s economic benefits, motivating enterprises to proactively improve their ESG performance. On the other hand, stakeholders’ preference for ESG in decision-making processes encourages enterprises to actively pursue ESG initiatives [4,5], thereby earning stakeholder trust and improving their business outcomes. For instance, governments and investment institutions can provide external financial support to enterprises, which not only ensures financial security for their ESG initiatives [6,7], but also fosters enterprise enthusiasm and encourages them to actively fulfill their social and other ESG responsibilities to secure further funding [3]. Overall, given the potential influence of external stakeholders on corporate ESG construction, it is crucial to examine how media attention, financing constraints, and government subsidies impact corporate ESG performance.

This study focuses on two aspects of the influencing process. Firstly, green technology innovation is a strategic tool that assists enterprises in integrating social and economic benefits, thereby promoting sustainable development [8]. As such, benefit drivers may impact corporate ESG performance through their influence on green technology innovation. Factors such as media attention, which serves a supervisory role [9], financing constraints and government subsidies, which directly impact a company’s internal funds [10,11], all contribute to the development of green technology innovation activities and research. This, in turn, significantly affects the progression of enterprise ESG construction [12].

Secondly, as corporate stakeholders increasingly prioritize companies with strong ESG performance and sustainable development capabilities, peer pressure may mitigate the influence of benefit drivers on green technology innovation. Companies under heightened peer pressure are likely to engage more actively in green technology innovation to maintain their competitive advantage, especially in the context of media attention, which has both reputation management and supervisory functions [13]. Moreover, investors and governments tend to favor enterprises with good corporate ESG performance [14]. To avoid investment risks, these entities may reduce their trust and financial support in companies under greater pressure, thus making it more challenging for these companies to secure sufficient funds for green technology innovation activities.

This study examines the impact of various benefit drivers - media attention, financing constraints, and government subsidies - on the Environmental, Social, and Governance (ESG) performance of 724 listed companies in China from 2015 to 2021. It delves into the mediating role of green technology innovation and the moderating influence of peer pressure. The research contributes in two primary ways: Firstly, it provides a stakeholder-centric perspective on how the involvement and preferences of external stakeholders can influence a company’s ESG performance. This systematic exploration of benefit drivers enriches existing research on factors that impact corporate ESG performance. Secondly, the study sheds light on the mediating effect of green technology innovation and the moderating effect of peer pressure, demystifying the mechanisms by which benefit drivers exert their influence on ESG performance. It establishes a pathway for how benefit drivers can influence corporate ESG performance through the lens of green technology innovation, and explores the moderating role of peer pressure in this process. Furthermore, the study provides practical guidance for companies to balance social and economic benefits, contributing to sustainable development. It also offers a theoretical reference for society and nation-states to guide companies in implementing ESG concepts.

The remainder of this study is organized into several key sections. Firstly, section 2 provides a detailed theoretical analysis and establishes the research hypotheses. This is followed by section 3, where the methodologies employed and the data utilized are outlined. In section 4, the empirical results are presented and subsequently discussed. Section 5 delves into the outcomes of the heterogeneity analysis. The study concludes with a comprehensive summary of the principal research findings and their pertinent policy implications.

## 2. Theoretical analysis and hypothesis development

### 2.1. Multiple benefit drivers and corporate ESG performance

#### 2.1.1. Media attention and corporate ESG performance.

The media, serving as a crucial information intermediary, aids in the conveyance of social dynamics and interest demands from various constituencies to businesses. It also assists these enterprises in garnering and maintaining support from stakeholders by disseminating relevant information to the external world. The influence of media attention on corporate ESG performance primarily hinges on two factors: firstly, the media, along with the general public, are significant stakeholders in the ESG construction process. As per stakeholder theory, enterprises and other organizations need to engage and communicate proactively with their stakeholders to accomplish their objectives [15]. Consequently, the media’s predilection for ESG prompts enterprises to prioritize their ESG construction. Those under media scrutiny are compelled to enhance their corporate ESG performance to ensure positive reporting, thereby facilitating the attainment of their business goals. For instance, when a company garners significant media attention, it often strives to augment positive news reports, cultivate a favorable corporate image, and secure the trust and support of external stakeholders. As a result, the company is incentivized to bolster its environmental governance and diligently meet its social responsibilities [16]. Furthermore, from the perspective of signal theory, the media serves to bridge the information gap between companies and their external stakeholders. They act as vital channels for disseminating ESG-related data to the general public through various reports [9]. Moreover, the media exert a supervisory function by ensuring that ESG disclosures adhere to regulatory norms, thus urging enterprises to improve their ESG performance. Research has underscored that media scrutiny spotlights corporate environmental challenges in its reports, pushing companies to prioritize sustainable technologies and address environmental governance issues [17]. Consequently, in response to heightened media attention, companies are more inclined to actively refine their governance practices and bolster their ESG performance. Hence, we propose that:

Hypothesis H1: There is a positive relationship between media attention and corporate ESG performance.

#### 2.1.2. Financing constraints and corporate ESG performance.

Financing constraints reflect the availability of external investment and internal capital flows for a company. According to stakeholder theory, investment institutions, as significant external stakeholders within an enterprise, exert influence over its internal capital flow. Consequently, financing constraints emerge as a critical determinant in the operational and developmental activities of the enterprise. The impacts of financing constraints on corporate ESG performance are twofold: Firstly, enterprises with varying financing constraints possess differing amounts of funds and conditions for ESG construction. Those with fewer financing constraints have sufficient funds for various operations and can more effectively carry out ESG activities. Conversely, those with more financing constraints have limited capital flow to undertake ESG construction, which impedes the improvement of their corporate ESG performance. Secondly, to mitigate investment risks, investors supervise various business activities of enterprises, pay greater attention to ESG information, and urge enterprises to implement ESG construction. As capital providers and shareholders of enterprises, external investment institutions are important stakeholders of enterprises. In investment decision-making, the sustainable development capabilities and social responsibility of enterprises are highly valued in order to mitigate investment risks [18]. Corporate ESG performance is seen as a significant representation of these sustainable development capabilities. Consequently, investment institutions often exhibit strong ESG preferences in their investment decisions [19]. Specifically, enterprises with fewer financing constraints should actively strive to enhance their corporate ESG performance to meet investors’ ESG preferences, thereby securing ongoing trust and sustained financial support from investors. In summary, financing constraints can influence corporate ESG performance. Therefore, we propose that:

Hypothesis H2: There is a negative correlation between financing constraints and corporate ESG performance.

#### 2.1.3. Government subsidies and corporate ESG performance.

Government subsidies not only provide a significant source of external funding for enterprises but also serve as a key mechanism to steer enterprise responses in line with national policies. As a crucial external stakeholder, the government wields substantial influence over an enterprise’s business activities, per stakeholder theory. Therefore, it is vital for enterprises to take into account the needs and expectations of these stakeholders for more effective goal attainment. Consequently, government support and aid play pivotal roles in shaping corporate ESG performance. The impact of government subsidies on corporate ESG performance can be twofold. Firstly, they provide adequate funding for enterprises to undertake ESG initiatives, including active environmental management and social responsibility. Secondly, as a key initiator of ESG, the government uses financial subsidies to incentivize and guide enterprises towards improved corporate ESG performance. Besides, the enterprises receiving government financial support are better positioned to fund green innovation and enhance environmental governance, thereby improving their corporate ESG performance [20]. Conversely, companies receiving government subsidies are more likely to adhere to government directives, adopt ESG principles, and improve their ESG performance. The government employs various strategies based on incentive theory to foster sustainable development within enterprises. Among these, government subsidies are the most common incentives, serving as a catalyst for corporate health and growth [21]. Through financial support, the government motivates companies to align with national policies. As a result, companies are more inclined to strengthen their ESG performance to maintain the trust and support of the government. Therefore, we propose that:

Hypothesis H3: There is a positive correlation between government subsidies and corporate ESG performance.

### 2.2. Mediating effect of green technology innovation

#### 2.2.1. Multiple benefit drivers and green technology innovation.

Green technology innovation serves as a pivotal tool for enterprises to achieve sustainable development and enhance their corporate ESG performance. Based on stakeholder theory, it is clear that primary stakeholders such as the media, investment institutions, and government significantly influence an enterprise’s green technology innovation activities. Such influence can be exerted through various means like media attention, financing constraints, and government subsidies. Primarily, media attention can encourage enterprises to focus more on stakeholder needs, guide them to prioritize ecological environmental protection, and motivate them to enhance their performance in green technology innovation [22,23]. Secondly, due to the high risk associated with green technology innovation activities and considerable dependence on capital, enterprises facing financing constraints bear high financing costs. Consequently, they struggle to secure adequate funds from external sources for these activities, thereby limiting their green technology innovation development [24]. Lastly, government subsidies serve as an essential measure for encouraging enterprises’ research and technology development. These subsidies motivate enterprises to address environmental issues, boost investment in research and development, promote green transformation, and stimulate the implementation of green technology innovation [25]. This has a positive impact on an enterprise’s green innovation activities. In conclusion, benefit drivers significantly influence an enterprise’s green technology innovation. Thus, we posit that:

Hypothesis H4: There is a correlation between benefit drivers and green technology innovation.

Hypothesis H4a: There is a positive correlation between media attention and green technology innovation.

Hypothesis H4b: There is a negative correlation between financing constraints and green technology innovation.

Hypothesis H4c: There is a positive correlation between government subsidies and green technology innovation.

#### 2.2.2. Multiple benefit drivers, green technology innovation and corporate ESG performance.

Innovation theory attests to the pivotal role of innovation in driving corporate economic growth and bolstering competitiveness. Innovation equips companies with the tools to secure competitive advantages, foster technological advancements, and markedly improve performance across diverse sectors [26]. Green technology innovation serves as a crucial nexus for aligning economic impacts with social benefits, also acting as a significant avenue to enhance corporate ESG performance. This innovation not only augments sustainable competitiveness but also facilitates sustainable development while aiding in the enhancement of corporate ESG metrics. Initial findings suggest that by strengthening their green technology innovation capabilities, corporations can optimize pollutant management, augment environmental advantages, and enhance their environmental governance [27]. Additionally, the pursuit of research and development in novel technologies enables companies to better fulfill their social obligations, thereby elevating their social responsibility metrics [28]. Moreover, heightened levels of corporate green technology innovation contribute to a favorable corporate image, diminish investment risks and equity capital costs, and refine corporate governance structures [29]. Thus, the corporate ESG performance can be enhanced through various facets: environmental, social responsibility, and corporate governance. The evidence underscores the beneficial impact of green technology innovation on corporate ESG metrics. Given that certain drivers influence green technology innovation, this study suggests that factors such as media attention, financial constraints, and government subsidies can shape green technology innovation endeavors, subsequently impacting corporate ESG performance. Thus, we posit that:

Hypothesis H5: Green technology innovation plays a mediating effect between benefit drivers and corporate ESG performance.

Hypothesis H5a: Green technology innovation plays a mediating effect between media attention and corporate ESG performance.

Hypothesis H5b: Green technology innovation plays a mediating effect between financing constraints and corporate ESG performance.

Hypothesis H5c: Green technology innovation plays a mediating effect between government subsidies and corporate ESG performance.

### 2.3. Moderating effect of peer pressure

According to dynamic competition theory, businesses operate within a continually competitive environment and are subject to pressures from their peers. In order to gain a competitive edge and secure external resources, it is imperative for enterprises to engage in ongoing innovation and improvement [30]. Similarly, companies compete fiercely for a range of resources provided by stakeholders. The influence of peer pressure may alter the impact of benefit drivers - such as media attention, financial constraints, and government subsidies - on green technology innovation. In one respect, peer pressure can amplify the positive effect of media attention on green technology innovation. Specifically, as media fulfills a supervisory role and contributes to reputational governance, it has the power to influence corporate reputation. Consequently, companies under significant peer pressure are keen to cultivate a positive image for stakeholders via media coverage. When faced with media scrutiny, these firms become more motivated to pursue green technology innovation, in order to maintain their competitive edge by developing new technologies, thereby earning the trust of stakeholders and increasingly engaging in innovative projects. However, in another respect, peer pressure can intensify the negative impact of financial constraints on green technology innovation while diluting the positive influence of government subsidies on the same. Notably, since government bodies, investors, and other stakeholders have established ESG preferences, they tend to place greater trust in companies demonstrating a commitment to green innovation and sustainable development [31]. Concurrently, firms may experience a reduction in financial support if they are subject to increased scrutiny from their peers. As a result, companies that face heightened peer pressure—even those that have previously secured financial backing from investors and the government—might be apprehensive about the potential discontinuation of such support or the challenges of ensuring long-term sustainability. Consequently, without substantial investments in green technology innovation, the propensity of enterprises to undertake these initiatives might wane. Thus, we posit that:

Hypothesis H6: Peer pressure moderates the correlation between benefit drivers and green technology innovation.

Hypothesis H6a: Peer pressure positively moderates the positive correlation between media attention and green technology innovation.

Hypothesis H6b: Peer pressure positively moderates the negative correlation between financing constraints and green technology innovation.

Hypothesis H6c: Peer pressure negatively moderates the positive correlation between government subsidies and green technology innovation.

## 3. Methodology and variable definitions

### 3.1. Methodology

This study analyzed 724 heavily polluting companies listed in China from 2015 to 2021. The chosen data, collected before the outbreak, boasted high availability and completeness, thereby ensuring reliable and valid research results. After initial data processing, firms marked as ST or *ST, along with samples featuring missing data values or outliers, were excluded. This resulted in a final dataset of 5068 sample observations. The ESG rating data was sourced from the Wind database, while media attention and green technology innovation data were obtained from the CNRDS database. The majority of the other data was collected from the CSMAR database. All data processing was conducted using Stata16.0 software.

### 3.2. Variable definition

#### 3.2.1. Dependent variable.

Corporate ESG Performance (ESG): Drawing on the research of scholars such as Ning and Zhang (2023), this study employs the Hua Zheng ESG Rating Index, which is constructed using publicly available information and data from the Wind database [32]. This comprehensive rating covers a broad spectrum and integrates China’s unique national conditions by amalgamating foreign ESG rating databases. The rating system is tiered into nine levels, with scores ranging from 1 to 9, reflecting the lowest to highest performance. Ratings are issued quarterly, and the average of the four annual ratings serves as the proxy variable for corporate ESG performance.

#### 3.2.2. Independent variables.

Media Attention (MA): This paper adopts the approach of Chai et al. (2023) by selecting the quantity of online and newspaper media coverage from the CNRDS database. The natural logarithm of the total number of media reports is then calculated to serve as the proxy variable for media attention [33].

Financing Constraints (FC): This study employs the methodology proposed by Hadlock and Pierce (2010) for measuring financing constraints. It constructs the SA index using the age and size of enterprises, and uses the absolute value of this index as the proxy variable for financing constraints [34]. A larger absolute value indicates more severe financing constraints for enterprises.

Government Subsidies (GS): Following the research of Wu and Hu (2020), this study uses the natural logarithm of the government subsidies received by enterprises in the current year as the proxy variable for government subsidies [35].

#### 3.2.3. Mediating variable.

Green Technology Innovation (GI): This study utilizes the number of green patent applications from enterprises as a proxy variable for their green technology innovation efforts. This measure reflects the extent to which companies are engaging in environmentally friendly technological advancements, a perspective supported by researchers such as Ma et al. (2021) [36].

#### 3.2.4. Moderating variable.

Peer Pressure (PR): As indicated by researchers such as Cao et al. (2018), peer enterprises are those that belong to the same enterprise network as the focus enterprises [37]. Within this network, beyond the focus enterprises themselves, the average ESG performance of these peer enterprises serves as a proxy variable for measuring the peer pressure exerted on the focus enterprises. A higher average value suggests greater peer pressure on the focus enterprises.

#### 3.2.5. Control variables.

In accordance with prior research, this study has controlled for variables that could potentially influence corporate ESG performance. The following control variables were selected: debt-to-liability ratio (Debt), return on total assets (Roa), company age (Age), growth capability (Growth), current asset ratio (Tangi), and board size (Board). Additionally, industry dummy variables (Industry) and time dummy variables (Year) were incorporated. These variables are detailed in Table 1.

**Table 1:** Definition and measurement of variables.

Variables	Code	Measurement
Dependent variable	ESG	The average of the four ratings of Hua Zheng ESG Rating Index score.
Independent variables	MA	The natural logarithm of total media coverage.
	FC	Absolute value of the SA index.
	GS	The natural logarithm of government subsidy.
Mediating variable	GI	Number of green patent applications.
Moderating variable	PR	The average ESG performance of the same group of enterprises in the network, except the focus enterprises.
Control variables	Debt	Total liabilities/ Total assets of enterprise.
	Roa	net profit/ Total assets of enterprise.
	Age	The number of years the company has been listed.
	Growth	Growth rate of main business income.
	Tangi	Current assets/ Total assets of enterprise.
	Board	The total number of board members.
	Ind	Industry variable.
	Year	Year variable.

### 3.3. Model

This study aims to examine the impact mechanism of beneficial drivers, namely media attention, financing constraints, and government subsidies, on corporate ESG performance. For this purpose, a regression model, designated as Model 1, has been formulated, as illustrated in Equation (1). In this model, the dependent variable represents corporate ESG performance, while the independent variables encompass media attention, financing constraints, and government subsidies. The regression model is:


$$ESG_{i,t}=\alpha_{0}+\alpha_{1}X_{i,t}+\alpha_{2}Controls+\sum_{t=1}^{m}year_{t}+\sum_{i=1}^{n}ind_{i}+\varepsilon_{i,t}$$ (1)

This study aims to confirm the mediating effect of green technology innovation, for which it has formulated Models 2 and 3, depicted in Equations (2) and (3). In these models, GI represents green technology innovation. It is important to note that GI serves as the dependent variable in Model 2 and the mediating variable in Model 3. Additionally, the independent variable X encompasses media attention, financing constraints, and government subsidies. The regression model is:


$$GI_{i,t}=\beta_{0}+\beta_{1}X_{i,t}+\beta_{2}Controls+\sum_{t=1}^{m}year_{t}+\sum_{i=1}^{n}ind_{i}+\varepsilon_{i,t}$$ (2)

$$ESG_{i,t}=\beta_{0}+\beta_{1}X_{i,t}+\beta_{2}GI_{i,t}+\beta_{3}Controls+\sum_{t=1}^{m}year_{t}+\sum_{i=1}^{n}ind_{i}+\varepsilon_{i,t}$$ (3)

To evaluate the regulatory impact of peer pressure, this study formulated a model, represented as equation (4). In this model, the dependent variable GI represents green technology innovation. The independent variables, denoted as X, encompass media attention, financing constraints, and government subsidies. Additionally, the moderating variable PR is identified as peer pressure. The regression model is:


$$GI_{i,t}=\eta_{0}+\eta_{1}X_{i,t}+\eta_{2}GI_{i,t}*PR_{i,t}+\eta_{3}Controls+\sum_{t=1}^{m}year_{t}+\sum_{i=1}^{n}ind_{i}+\varepsilon_{i,t}$$ (4)

Within the regression model, “Controls” denotes the control variable, while “Year” and “Ind” respectively signify the time dummy variable and the industry dummy variable. Furthermore, ε characterizes the random error.

## 4. Data analysis and results discussion

### 4.1. Descriptive statistics

The descriptive statistical results are presented in Table 2. The mean value of corporate ESG performance (ESG) stands at 6.428 with a variance of 1.197, indicating significant disparities and uneven development in the ESG performance across different enterprises. The average media attention (MA) is 5.671, showcasing a variance of 1.479 and highlighting substantial variations in media coverage among enterprises. The mean value for financing constraints (FC) is 3.864 with a variance of 0.248, suggesting that while most enterprises face notable financing challenges, the differences between them are minimal. The average government subsidy deposit (GS) is 17.72, with a variance of 2.211, revealing marked variations in government subsidies received by different enterprises. Other variables’ descriptive statistics also fall within the normal range.

**Table 2:** Descriptive statistics of variables.

Variable	N	Mean	SD	MIN	MAX
ESG	5068	6.428	1.197	2	9
MA	5068	5.671	1.479	0	13.68
FC	5068	3.864	0.248	2.403	5.318
GS	5068	17.72	2.211	0	23.03
GI	5068	10.32	74.10	0	2,371
PR	5068	6.506	0.923	2	9
Roa	5068	0.163	0.344	−8.669	5.476
Debt	5068	0.424	0.192	0.001	1.515
Age	5068	19.44	5.555	6.330	54
Growth	5068	0.991	24.24	−9.997	1,142
Tangi	5068	0.914	0.0874	0.365	1
Board	5068	10.33	2.631	5	26

### 4.2. Correlation analysis

Table 3 presents the results of the correlation analysis among the primary variables. There is a significant positive correlation between the dependent variable, corporate ESG performance (ESG), and the independent variables, media attention (MA) and government subsidies (GS). Conversely, there is a significant negative correlation between ESG and financing constraints (FC). These findings provide preliminary support for the research hypothesis formulated in this study. The Variance Inflation Factor (VIF) test reveals that the VIF value for each variable in this study is considerably less than 10, thereby largely mitigating concerns about multicollinearity among the variables.

**Table 3:** Correlation analysis of main variables.

Variable	ESG	MA	FC	GS	GI	PR	VIF
ESG	1						
MA	0.197***	1					1.13
FC	−0.055***	−0.091***	1				1.09
GS	0.179***	0.252***	−0.049***	1			1.07
GI	0.118***	0.230***	−0.190***	0.113***	1		1.04
PR	0.132***	0.104***	−0.0120	0.0160	0.047***	1	1.01

t-statistics in parentheses*** p< 1%, ** p < 5%, * p < 10%.

### 4.3. Regression results and analysis

#### 4.3.1. Multiple benefit drivers and Corporate ESG performance.

In this study, we utilized Stata16.0 to assess the influence of benefit drivers on corporate ESG performance. Through a benchmark regression analysis of model (1), the results presented in Table 4 were obtained. These results reveal that the coefficients of media attention (MA), financing constraints (FC), and government subsidies (GS) on corporate ESG performance (ESG) are 0.205, −1.908, and 0.093, respectively. Notably, these coefficients are significant at the 1% level. Our findings suggest that media attention positively affects corporate ESG performance, thus supporting H1; financing constraints negatively impact corporate ESG performance, thereby supporting H2; and government subsidies positively influence corporate ESG performance, endorsing H3. Furthermore, it is evident that increased media attention, reduced financing constraints, and augmented government subsidies are associated with enhanced corporate ESG performance. Among the benefit drivers, financing constraints exert the most pronounced effect on enterprises. This implies that investment institutions’ inclination towards corporate ESG performance and their influence on internal capital flows play a pivotal role in motivating enterprises to enhance their ESG performance.

**Table 4:** Benefit drivers and corporate ESG performance.

Variable	(1) ESG	(2) ESG	(3) ESG
MA	0.205***		
	(14.36)		
FC		−1.908***	
		(−14.77)	
GS			0.093***
			(12.36)
Controls	YES	YES	YES
Year PE	YES	YES	YES
Ind PE	YES	YES	YES
Constant	4.166***	11.111***	3.730***
	(15.53)	(23.49)	(13.02)
N	5068	5068	5068
R^2^	0.147	0.148	0.138
P	0.000	0.000	0.000

① t-statistics in parentheses *** p < 1%, ** p < 5%, * p < 10%. ② In order to save space, the results of control variables are not reported.

#### 4.3.2. The mediating effect of green technology innovation.

Firstly, a benchmark regression test was administered on model (2) to examine the relationship between benefit drivers and green technology innovation. The results of this regression are presented in columns (1), (3), and (5) of Table 5. These results reveal that the regression coefficients of media attention (MA), financing constraints (FC), and government subsidies (GS) on green technology innovation (GI) are 12.203, -192.903, and 2.815 respectively. Each of these coefficients is statistically significant at the 1% level. Consequently, it can be concluded that media attention and government subsidies significantly positively influence green technology innovation, while financing constraints significantly negatively impact it, thereby providing support for hypothesis H4.

**Table 5:** The mediating effect of green technology innovation.

Variable	(1) GI	(2) ESG	(3) GI	(4) ESG	(5) GI	(6) ESG
MA	12.203***	0.187***				
	(14.29)	(12.99)				
FC			−192.903***	−1.739***		
			(−25.72)	(−12.66)		
GS					2.815***	0.088***
					(6.12)	(11.75)
GI		0.001***		0.001***		0.002***
		(5.69)		(3.59)		(7.35)
Controls	YES	YES	YES	YES	YES	YES
Year PE	YES	YES	YES	YES	YES	YES
Ind PE	YES	YES	YES	YES	YES	YES
Constant	−89.564***	5.169***	567.455***	11.791***	−71.160***	4.588***
	(−5.53)	(11.07)	(20.67)	(18.53)	(−4.06)	(9.55)
N	5068	5068	5068	5068	5068	5068
R^2^	0.185	0.152	0.251	0.150	0.158	0.147
P	0	0	0	0	0	0

t-statistics in parentheses ^***^ p < 1%, ^**^ p < 5%, ^*^ p < 10%.

Secondly, a benchmark regression test was conducted on model (3), and the results are displayed in columns (2), (4), and (6) of Table 5. After incorporating the mediating variable of green technology innovation, the regression coefficients of media attention (MA), financing constraints (FC), and government subsidies (GS) on corporate ESG performance were found to be 0.187, -1.739, and 0.088 respectively. These coefficients are statistically significant at the 1% level. Notably, these regression coefficients are smaller than those presented in Table 4, where no mediating variable was included. The regression results in Table 5 also reveal that the coefficients of the mediating variable, green technology innovation, on corporate ESG performance are 0.001, 0.001, and 0.002 respectively, all demonstrating significance at the 1% level. To summarize, these findings suggest that green technology innovation partially mediates the impact of media attention, financing constraints, and government subsidies on corporate ESG performance, thereby supporting hypothesis H5. This indicates that these factors can influence corporate ESG performance through their effect on corporate green technology innovation.

#### 4.3.3. The moderating effect of peer pressure.

A baseline regression test was conducted on model (4) to examine the moderating effect of peer pressure, with the results presented in Table 6. Upon reviewing columns (2), (4), and (6) of Table 6, it is evident that the regression coefficient for the interaction terms (MA*PR), which represents the relationship between media attention (MA) and peer pressure (PR) on green technology innovation (GI), is 2.998. This coefficient is statistically significant at the 1% level. Conversely, the regression coefficient for the interaction terms (FC*PR), indicating the relationship between financing constraints (FC) and peer pressure (PR) on GI, is -18.948, and this too is significant at the 1% level. Additionally, the regression coefficient for the interaction terms (GS*PR), representing the impact of government subsidies (GS) and peer pressure (PR) on GI, is −0.923. This coefficient is significant at the 10% level. It should be noted that the individual regression coefficients for media attention, financing constraints, and government subsidies on green technology innovation are 11.577, −186.742, and 2.979 respectively. The coefficients presented are statistically significant at the 1% level. Upon integrating the two sets of coefficients, it is inferred that peer pressure has a pronounced positive effect on the favorable influence of media attention regarding green technology innovation, as well as on the adverse impact of financing constraints related to green technology innovation. Concurrently, it significantly diminishes the positive effect of government subsidies on green technology innovation, given that both H6 are upheld. This indicates that increased peer pressure bolsters a company’s dedication to pursuing green technology innovation endeavors, especially when such media attention serves to enhance the company’s public reputation. As a result, peer pressure can potentiate the beneficial impact of media attention on green technology innovation. Nevertheless, it is pertinent to mention that excessive peer pressure might precipitate a withdrawal of financial support from investors and governments, subsequently impeding the implementation of innovative endeavors. Hence, it may mitigate the favorable effects of financing constraints and government subsidies on green technology innovation.

**Table 6:** The moderating effect of peer pressure.

Variable	(1) GI	(2) GI	(3) GI	(4) GI	(5) GI	(6) GI
MA	12.113***	11.577***				
	(14.16)	(13.41)				
MA*PR		2.998***				
		(4.26)				
FC			−184.969***	−186.742***		
			(−24.27)	(−24.60)		
FC*PR				−18.948***		
				(−4.62)		
GS					2.789***	2.979***
					(6.06)	(6.31)
GS*PR						−0.923*
						(−1.76)
PR	1.772*	1.764*	2.049**	1.975*	2.579**	2.697**
	(1.65)	(1.65)	(2.00)	(1.92)	(2.37)	(2.47)
Controls	YES	YES	YES	YES	YES	YES
Year PE	YES	YES	YES	YES	YES	YES
Ind PE	YES	YES	YES	YES	YES	YES
Constant	−31.098**	−30.123*	−185.720***	−184.318***	−32.112**	−31.503**
	(−2.00)	(−1.94)	(−11.65)	(−11.49)	(−2.03)	(−1.99)
N	5068	5068	5068	5068	5068	5068
R^2^	0.186	0.189	0.246	0.254	0.159	0.160
P	0	0	0	0	0	0

t-statistics in parentheses^***^ p < 1%, ^**^ p < 5%, ^*^ p < 10%.

### 4.4. Robustness

#### 4.4.1. Lag on the independent variable.

To address potential endogeneity among variables, a one-phase lag processing approach was employed. Consequently, the independent variable for the t period was adjusted to the t-1 period, and the analysis was re-run. The findings, presented in Table 7, indicate that media attention and government subsidies significantly positively influence corporate ESG performance. Conversely, financing constraints demonstrate a significant negative effect on corporate ESG performance. These outcomes align with prior research, suggesting that there is no inherent endogeneity among the variables, thereby affirming the robustness of the results.

**Table 7:** Benefit drivers and corporate ESG performance (Lag on the independent variable).

Variable	(1) ESG	(2) ESG	(3) ESG
MA_t-1_	0.209***		
	(11.71)		
FC_t-1_		−1.838***	
		(−12.72)	
GS_t-1_			0.076***
			(9.26)
Controls	YES	YES	YES
Year PE	YES	YES	YES
Ind PE	YES	YES	YES
Constant	4.014***	10.791***	3.896***
	(13.50)	(20.54)	(12.44)
N	4344	4344	4344
R^2^	0.146	0.150	0.135
P	0.000	0.000	0.000

t-statistics in parentheses^***^ p < 1%, ^**^ p < 5%, ^*^ p < 10%.

#### 4.4.2. Replace the measurement of mediating variable.

This study employed a method of replacing the mediating variable in measurement, utilizing the count of green invention patents as a substitute for the original measurement method to quantify green technology innovation. A robustness test was conducted to ensure the stability of the research results. The regression results are presented in Table 8. Within the framework of the impact of benefit drivers on corporate ESG performance, green technology innovation serves as a mediating element. This finding aligns broadly with those of previous studies, thereby reinforcing the robustness of the results obtained in this study.

**Table 8:** The mediating effect of green technology innovation (Replace the measurement of mediating variable).

Variable	(1) GI	(2) ESG	(3) GI	(4) ESG	(5) GI	(6) ESG
MA	5.835***	0.187***				
	(12.89)	(13.08)				
FC			−117.462***	−1.739***		
			(−30.30)	(−12.38)		
GS					0.954***	0.090***
					(3.91)	(11.99)
GI		0.003***		0.001***		0.003***
		(6.14)		(3.05)		(7.93)
Controls	YES	YES	YES	YES	YES	YES
Year PE	YES	YES	YES	YES	YES	YES
Ind PE	YES	YES	YES	YES	YES	YES
Constant	−43.507***	5.170***	348.144***	11.532***	−28.290***	4.585***
	(−5.06)	(11.08)	(24.53)	(17.81)	(−3.05)	(9.56)
N	5068	5068	5068	5068	5068	5068
R^2^	0.322	0.153	0.407	0.150	0.301	0.148
P	0	0	0	0	0	0

t-statistics in parentheses^***^ p < 1%, ^**^ p < 5%, ^*^ p < 10%.

#### 4.4.3. System generalized method of moments.

This study addresses the issue of endogeneity, particularly the bidirectional causal relationship between independent and dependent variables, by utilizing the System Generalized Method of Moments (System GMM). This method is widely recognized for its effectiveness in addressing endogeneity issues. It combines the difference system with the level system, using both the lag term of the independent variable and its difference term as instrumental variables, thereby enhancing the validity of the research findings. The test results are presented in Table 9. All research outcomes withstood both the Arellano-Bond and Hansen tests, leading to the acceptance of the null hypothesis of exogeneity of the instrumental variables. This confirms the reliability of the estimation results. The regression results align closely with previous findings, indicating no endogeneity issues within the variables explored in this study. Therefore, the research findings can be considered robust.

**Table 9:** Benefit drivers and corporate ESG performance (System GMM).

Variable	(1) ESG	(2) ESG	(3) ESG
MA	0.051**		
	(2.51)		
FC		−6.598***	
		(−3.10)	
GS			0.489***
			(2.64)
Controls	YES	YES	YES
Year PE	YES	YES	YES
Ind PE	YES	YES	YES
AR (1) test	0.000	0.000	0.028
AR (2) test	0.666	0.164	0.278
Hansen test	0.407	0.276	0.762
F test	0	0	0
N	4344	4344	4344

① t-statistics in parentheses ^***^p < 0.01, ^**^p < 0.05, ^*^p < 0.1. ② In order to avoid estimation bias caused by too many instrumental variables, the collapse option is added to control the instrumental variables during estimation. ③ AR(1)test、AR(2)test is the differential autocorrelation test of the disturbance term, Hansen test is an over-identification test.

## 5. Heterogeneity test

### 5.1. Heterogeneity analysis results of resource acquisition capability

The impact of benefit drivers on a corporation’s Environmental, Social, and Governance (ESG) performance can vary based on the enterprise’s resource acquisition capabilities. An enterprise cannot enhance its ESG performance without the appropriate resources such as knowledge and technology. Consequently, enterprises that can allocate resources for ESG development are in a better position to improve their ESG performance. This study further examines the varying impacts of benefit drivers on corporate ESG performance, taking into account the differing resource acquisition capabilities of enterprises.

Existing research has highlighted that the relationships formed between interlocking directors within management teams can facilitate the acquisition of advanced resources necessary for the enterprise [38]. Corporations positioned at the center of such networks exhibit a superior capacity for obtaining external resources. Consequently, this study employs the count of interlocking directors within the management as a metric for the enterprise’s resource acquisition capability. The sampled enterprises were categorized based on their resource acquisition capabilities in ascending order. The bottom third of the sampled enterprises were classified as having low resource acquisition capabilities, while the top third were classified as having high resource acquisition capabilities. Subsequent multiple regression analyses were conducted on these two groups. The findings, presented in Table 10, indicate the regression results for the high resource acquisition capability group in columns (1), (2), and (3). The regression coefficients for media attention (MA), financing constraints (FC), and government subsidies (GS) on corporate ESG performance (ESG) were found to be 0.217, −2.942, and 0.213 respectively. The coefficients presented in this study are statistically significant at the 1% level. Specifically, columns (4), (5), and (6) display the regression results for the group with low resource acquisition capabilities. The regression coefficients for media attention (MA), financing constraints (FC), and government subsidies (GS) on corporate ESG performance (ESG) are 0.202, 1.4695, and 0.093, respectively. All of these coefficients are significantly relevant at the 1% level. Comparing the two columns of regression coefficients reveals that for enterprises with high resource acquisition capabilities, factors such as media attention, financing constraints, and government subsidies exert a relatively larger impact on corporate ESG performance.

**Table 10:** Heterogeneity analysis results of resource acquisition capability.

Variable	Sample of high resource acquisition capability			Sample of low resource acquisition capability		
	(1) ESG	(2) ESG	(3) ESG	(4) ESG	(5) ESG	(6) ESG
MA	0.217***			0.202***		
	(9.51)			(7.74)		
FC		-2.942***			−1.469***	
		(-12.71)			(−6.23)	
GS			0.213***			0.093***
			(12.71)			(6.94)
Controls	YES	YES	YES	YES	YES	YES
Year PE	YES	YES	YES	YES	YES	YES
Ind PE	YES	YES	YES	YES	YES	YES
Constant	4.071***	14.013***	1.420**	4.552***	10.178***	4.206***
	(7.97)	(16.18)	(2.51)	(11.02)	(12.75)	(9.43)
N	1689	1689	1689	1689	1689	1689
R^2^	0.167	0.200	0.200	0.168	0.157	0.161
P	0.000	0.000	0.000	0.000	0.000	0.000

t-statistics in parentheses^***^ p < 1%, ^**^ p < 5%, ^*^ p < 10%.

### 5.2. Heterogeneity analysis results of ownership structure

Since the inception of aggressive calls for enterprises to adopt the ESG concept, the property rights of these entities may have been impacted, potentially affecting their ESG construction. This paper further delves into a heterogeneity analysis, predicated on the property rights of enterprises.

This study categorizes the sample data into two groups: state-owned enterprises and non-state-owned enterprises, conducting separate regression analyses for each. The results of these regression analyses are displayed in Table 11. From these results, it is evident that within state-owned enterprises, factors such as media attention, financing constraints, and government subsidies significantly influence the ESG performance of the enterprises.

**Table 11:** Heterogeneity analysis results of ownership structure.

Variable	State-owned enterprises			Non-State-owned enterprises		
	(1) ESG	(2) ESG	(3) ESG	(4) ESG	(5) ESG	(6) ESG
MA	0.249***			0.174***		
	(9.98)			(10.55)		
FC		−2.085***			−1.660***	
		(−9.83)			(−11.05)	
GS			0.129***			0.083***
			(8.58)			(9.42)
Controls	YES	YES	YES	YES	YES	YES
Year PE	YES	YES	YES	YES	YES	YES
Ind PE	YES	YES	YES	YES	YES	YES
Constant	3.743***	11.745***	2.950***	3.726***	10.051***	3.328***
	(11.26)	(15.35)	(7.62)	(15.22)	(18.75)	(12.33)
N	1,639	1,639	1,639	3,429	3,429	3,429
R^2^	0.117	0.115	0.103	0.108	0.111	0.102
P	0.000	0.000	0.000	0.000	0.000	0.000

t-statistics in parentheses^***^ p < 1%, ^**^ p < 5%, ^*^ p < 10%.

## 6. Conclusions

### 6.1. Research conclusions

This study conducted a theoretical analysis and empirical research to examine the relationship between benefit drivers (such as media attention, financing constraints, and government subsidies) and corporate Environmental, Social, and Governance (ESG) performance. It also evaluated the mediating impact of green technology innovation and the moderating influence of peer pressure. The findings reveal that:

Firstly, we discovered a positive correlation between media attention and government subsidies, and corporate ESG performance. Conversely, financing constraints showed a negative correlation with the same performance metrics. Among these drivers of benefits, financing constraints demonstrated the most substantial correlation with corporate ESG performance. In particular, firms that enjoy elevated media attention, diminished financing constraints, and augmented government subsidies tend to display greater enthusiasm and financial resources to boost their ESG performance, thus achieving superior levels of corporate ESG performance.

Secondly, our examination of the mediating role of green technology innovation revealed that it significantly mediates the relationship between benefit drivers (such as media attention, financing constraints, and government subsidies) and corporate ESG performance. Specifically, these drivers—media attention, financing constraints, and government subsidies—can shape corporate ESG performance by affecting the extent of green technology innovation. Thus, by enhancing their green technology innovation capabilities, enterprises can effectively augment their ESG performance. This enhancement in innovation capability can advance their environmental and corporate governance technologies, subsequently guiding them toward making sound social responsibility decisions, thereby elevating the overall corporate ESG performance.

Simultaneously, an analysis of the regulatory effects of peer pressure revealed that it can amplify the positive relationship between media attention and green technology innovation, as well as the negative relationship between financing constraints and green technology innovation. Conversely, it can diminish the positive correlation between government subsidies and green technology innovation. Specifically, when enterprises face high levels of peer pressure, they strive to cultivate a favorable corporate image and sustain competitive advantages via media coverage, thereby increasing their enthusiasm for enhancing green technology innovation. Consequently, peer pressure amplifies the positive correlation between media attention and green technology innovation. However, excessive peer pressure can erode an enterprise’s competitive edge and the trust and financial backing it receives from financial institutions and governments. This reduction in confidence about securing sufficient funds for innovative activities reduces the motivation of enterprises to improve green technology innovation. Therefore, peer pressure strengthens the negative correlation between financing constraints and green technology innovation, while weakening the positive correlation with government subsidies.

Finally, the results of the heterogeneity analysis reveal that in enterprises with robust resource acquisition capabilities, there is a stronger correlation between benefit drivers (such as media attention, financing constraints, and government subsidies) and corporate ESG performance. Essentially, the more resources an enterprise can acquire, the better it is equipped to reinforce its ESG construction. This subsequently amplifies the impact of benefit drivers on the corporation’s ESG performance, promoting enhanced progress in this area. Similarly, in state-owned enterprises, there exists a stronger correlation between benefit drivers and corporate ESG performance. State-owned enterprises are generally more responsive to national ESG policies and more capable of implementing ESG construction effectively. As a result, the influence of benefit drivers on their ESG performance is heightened, leading to substantial improvements in their ESG performance.

### 6.2. Managerial implications

Corporations must recognize the significance of external stakeholders to their Environmental, Social, and Governance (ESG) performance and should actively engage in ESG construction. Initially, corporations should seek to enhance their ESG performance in response to media coverage and supervision, using it as a tool to cultivate a positive corporate image and garner favor from external stakeholders. Additionally, they should consider the ESG preferences of investors, in order to gain their trust and secure sufficient external financial support. When receiving governmental subsidies and support, alignment with the ESG principles promoted by their respective governments is vital for corporations aiming for sustainable development. The development of green technology innovation should also be a priority, with corporations encouraged to foster new technologies to improve their ESG performance. Finally, learning from and emulating the best practices of peer corporations can help companies respond effectively to external pressure, facilitating collective progress within their industry.

It is imperative for governments, media outlets, and investors to recognize the significant role of external stakeholders, actively promote the ESG concept, and guide corporations towards improving their corporate social, environmental, and governance (ESG) performance. Firstly, the government should fully utilize its leading role in establishing the ESG system, developing a comprehensive framework, implementing relevant policies and programs, and providing guidance to businesses to adopt the ESG concept. Additionally, the government should allocate resources and funds to encourage businesses to implement ESG practices. Secondly, the media should function as corporate watchdogs, broaden the coverage of corporate ESG performance, and oversee corporate behavior. This would motivate companies to improve their ESG performance and guide governments, investors, and other stakeholders to prioritize ESG performance in their investment decisions. Lastly, investment institutions should consider an enterprise’s ESG performance when formulating investment strategies. This not only reduces their own investment risks but also encourages businesses to actively enhance their ESG performance.

### 6.3. Research limitations

This study has several limitations. First, its research sample exclusively comprises enterprises from heavily polluting industries. Future research should consider including samples from high-carbon industries to enhance the generalizability and applicability of the findings. Second, while this study explores the effects of media attention, financing constraints, and government subsidies on corporate ESG performance from a stakeholder perspective, it overlooks the role of corporate responsibility. It is plausible that companies might proactively enhance their ESG performance out of intrinsic responsibility. Future studies could examine other antecedent variables from this responsibility standpoint, such as network centrality and executive academic background. Third, this research focuses solely on the total volume of media attention, highlighting its positive correlation with corporate ESG performance. However, it is essential to note that negative media reports can potentially harm a company’s reputation, leading to investor withdrawal and customer loss. Such scenarios might compel companies to allocate resources toward public relations and image management instead of genuine ESG improvements, thereby engaging in “greenwashing” practices. Hence, a more nuanced examination of how both positive and negative media reports influence corporate ESG performance is warranted in future research. Lastly, this study predominantly focuses on the effects of green technology innovation and peer pressure and neglects other potential factors. Future investigations should delve into the roles of other elements within these influencing mechanisms, such as the mediating effects of digitization level or information transparency.

## Supporting information

S1 Data Thesis analysis data. It contains the core linear regression analysis data of this study.
(XLS)
